# Scoping Review of the Models for Case-Based Health Programs in Africa: Towards Case-Based Surveillance for HIV in Lesotho

**DOI:** 10.3390/ijerph23030308

**Published:** 2026-02-28

**Authors:** Maletsatsi E. Motebang, Puleng Ramphalla, Joyce Tsoka-Gwegweni

**Affiliations:** Department of Public Health, Faculty of Health Sciences, University of the Free State, Bloemfontein Campus, Bloemfontein 9300, South Africa; 2026004875@ufs4life.ac.za (P.R.); tsokagwegwenijm@ufs.ac.za (J.T.-G.)

**Keywords:** HIV and surveillance, case-based surveillance, patient-level health systems, infectious diseases surveillance, Lesotho and Africa

## Abstract

**Highlights:**

**Public health relevance—How does this work relate to a public health issue?**

- The scoping review is highly relevant as it emphasizes on the importance of establishing a case-based surveillance system especially for infectious diseases such as HIV in a low-income setting like Lesotho where the disease has a potential to spread if not control through proper systems.
- Having a case-based surveillance (CBS) system will help track disease patterns and identify hot spots for immediate intervention and allocation of resources. Strengthening surveillance will not only help with disease management but also with prevention and overall population health.

**Public health significance—Why is this work of significance to public health?**

- This study has a potential to transform Lesotho’s public health response by adopting and adapting proven models from other African countries.
- This study has the potential to ultimately strengthen Lesotho’s health system and provide a roadmap which can lead to sustainability.

**Public health implications—What are the key implications or messages for practitioners, policy makers and/or researchers in public health?**

- This study will help policy makers to ensure there are relevant data governance policies in place to ensure data security, confidentiality and privacy.
- Policy makers will prioritize the necessary steps to establish CBS such as having electronic medical records system, using open sources and using unique identifiers.

**Abstract:**

The review aims at exploring models for case-base health programs across Africa that could best help Lesotho succeed in its efforts to establish a case-based surveillance (CBS) system for their HIV program. The review involves looking through several sources and databases including EBSCOHOST, Google Scholar, Science Direct and PubMed. The insights of suitable models were from the following Africa countries: South Africa, Kenya, Guinea, Tanzania, Ghana, Mozambique and Zambia. The study articles were published within the last 10 years, specifically from 2014 to 2024. This range was used as part of their inclusion criteria to ensure relevance of the articles. The studied models focused on infectious diseases such as measles, HIVand COVID-19. The key takeaway is that setting up electronic medical records systems (EMRs) is critical as a first step for any effective CBS. Using unique identifiers, establishing clear data governance policies and building strong infrastructure is a necessity in making CBS work. For a successful establishment of CBS, Lesotho should adopt these strategies that can be sustainable, improve disease tracking, response and ultimately health outcomes for Basotho.

## 1. Introduction

Case-based surveillance system is critical in public health; it provides an understanding of diseases and how they spread and eventually helps determine appropriate interventions to control outbreaks [1].

Globally, HIV remains a public health concern with approximately 39.9 million people living with HIV (PLHIV) at the end of 2023 [2]. Out of all PLHIV, about 5% constitutes children 0–14 years while adults 15 and above years accounted for the remaining 95%. Of all WHO regions, Africa accounts for the highest proportion; 65% of PLHIV globally followed by south-east Asian region and region of the Americas each attributing 10% of PLHIV [2].

Additionally, HIV prevalence varies significantly by country, especially in Africa. In 2023, the top five countries with a high HIV prevalence in the world were Eswatini at 27.5%, Lesotho 20.5%, Botswana 19.7%, South Africa 16.6% and Mozambique 11.8% [3]. Although the countries experience high HIV rates, the antiretroviral treatment (ART) significantly improved the lives of many and resulted in reduced new infections [3].

Lesotho, as one of the African countries with high HIV burden, 20.5% among adults aged 15 and above has significantly made efforts to improve health outcomes but the country still experiences barriers such as limited infrastructure, inadequate resources and gaps that hinder timely and accurate collection data [3,4]. Again, there is limited capacity and training among health workers and gaps in policies to ensure data security and patient confidentiality [4,5].

### 1.1. Global Interventions for a Successful HIV CBS

As countries continue to manage HIV, there are many interventions put in place including the use of technology for easier programming. The Department of Affairs (VA) in North America attests to health data being more manageable with the use of technology for activities such as Veterans Aging Cohort Study Index (VACSs Index) which has helped in predicting all-cause mortality, cause-specific mortality and other health outcomes specifically for PLHIV [6]. For the HIV population monitoring, WHO recommended the establishment of CBS, which is not only useful for retaining individual-level data for everyone diagnosed with HIV but also for linkage between systems using unique identification number (UID) and retaining data gathered from multiple sources [4].

Accordingly, the importance of the UID for a successful CBS cannot be over-emphasized. Thailand uses UID to link pivotal databases in the country for effective patient management [7]. Benefits of the CBS include improved turnaround time for laboratory results and facilitation of reimbursements where necessary. However, the system only caters for nationals and excludes migrants in Thailand [7].

In 2016, France developed a mandatory surveillance system called SurCeGIDD of which CeGIDDs covers sexually transmitted infections (STIs) clinics. The system uses individual data aimed at guiding STI prevention programs and policies at national and sub-national levels [8]. The mandatory surveillance covers the prevalence of STIs but other key elements such as socioeconomic determinants that helped guide the development of policies and evaluation of service delivery. Prior to the use of SurCeGIDD, case detection was low, but the use of the system resulted in improved records from 2414 in 2017 to 382,890 in 2018 [8].

There are also notable successes in Africa of a successful surveillance system. Botswana implemented a system that uses national unique identification and insurance number to access health HIV services including HIV and other social services. This system enables easy access and linkages to HIV and other health services across health facilities within the country [7].

### 1.2. Challenges of Existing Health Systems for HIV Program in Africa

Despite the successes of CBS, especially in developed countries, Africa continues to encounter challenges hindering smooth implementation. A cross-sectional study on the performance of COVID-19 case-based surveillance system in the Federal Capital Territory (FCT), Nigeria, conducted by [9] highlighted some challenges as inadequate resources such as transport for response activities, data for internet which is for the system’s functionality, non-harmonization of the treatment protocol and high dependence on donor support which limits long term sustainability, to mention but a few [9].

Additionally, a situational assessment of data systems conducted in Tanzania, South Africa and Kenya highlighted a few challenges of existing health systems particularly for the HIV program. In Tanzania, one of the challenges is the unavailability of UID, which limits linkage to care after diagnosis [4]. Also, the country experienced infrastructure difficulties. “Various information technology issues were observed, including smaller facilities not having computers, inconsistent connectivity and power outages, and a lack of interoperability between the patient monitoring systems (PMS) and other data systems at the health facility” [10] (p. 4). Thus, for the successful implementation of CBS, the country must address the highlighted challenges and strengthen data quality while also enforcing adherence to and optimal use of standard operational procedures (SOPs).

With South Africa, Harklerode et al. revealed that the country has implemented a national PMS, which collects individual-level data at facility level although it is not available nationwide. Unlike Tanzania, South Africa uses a national identity number as the UID, although not assigned to about 10% of non-citizens residing in the country. However, the country is developing a health patient registration system, which will incorporate every patient seeking health services [4]. Nevertheless, South Africa challenges included staff shortages and over-populated health facilities resulting in non-adherence to SOPs, thereby limiting data completeness and quality.

Lastly, in Kenya, the assessment revealed that there were several data systems from which we can obtain individual-level data for HIV CBS. However, many facilities still use paper-based systems and EMR is only used at facilities with patients greater than 500. “Existing infrastructure presents challenges to capturing and storing individual-level data electronically. All visited facilities reported having experienced periodic power outages, and only larger facilities reported having backup generators” [4] (p. 5). Regarding the use of UID, the study assigned patients a unique number at facility level which is included in the PMS but cannot be transferred across facilities. This limits the accuracy of data matching and de-duplication process [4]. Lastly, due to a high burden of HIV and other infectious diseases in Africa, a well-functioning CBS is important for successful programming.

Ayanore et al. [11] conducted a systematic review, which revealed that surveillance systems improve clinical care, patients’ outcomes and health systems preparedness for public health threats. Thus, it is critical for African countries to have a stable CBS, which will routinely and systematically monitor HIV patients from the time of diagnosis and throughout their clinical care.

### 1.3. Aim and Objectives

The aim of the study is to review existing models for the case-based health programs in Africa that inform the development and implementation of case-based surveillance of HIV in Lesotho. The summary of the scoping review will include a description of studies; types of models or systems used and lastly challenges and successes. The specific objectives are:

(1) To identify and describe the existing models for case-based health programs in Africa.
(2) To determine the successes and challenges to implementation of these models in Africa.
(3) To assess about needs for development and implementation of a case-based surveillance and to recommend a model for Lesotho.
(4) To identify any gaps in the models that inform the development of a case-based surveillance for HIV in an African setting.

## 2. Materials and Methods

### 2.1. The Study Design

Using Preferred Reporting Items for Systematic Reviews and Meta analysis (PRISMAs), the study conducted a scoping review of documents and provided a guideline for reporting systematic reviews [12].

### 2.2. The Database Search

The study used several databases like EBSCOHOST, Science Direct and PubMed to search for articles. We used the following keywords: HIV and surveillance, case-based surveillance, patients-level health systems, infectious diseases surveillance, Lesotho and Africa to search for documents. The inclusion criteria included the articles related to implementation of case-based surveillance for infectious diseases in Africa not older than 10 years (2014–2024), relevant studies written in English, and both quantitative and qualitative, to assess the differences and results when implementing CBS. However, the authors included 1 article about “Trauma cases” [13]. On the other hand, the exclusion criteria covered articles published before 2014, those that did not focus of case-based surveillance or infectious diseases, articles not written in English and full texts that could not be accessed.

There were two reviewers involved in screening articles and full-text reviews. Selection bias minimized by ensuring that reviewers screened articles independently and reached a consensus where there were disagreements. The selection and screening of articles was through Rayyan “https://rayyan.qcri.org (accessed on 24 March 2025)”, a tool useful for both literature and systematic reviews.

### 2.3. Inclusion of Data Sources

The study used PRISMA method for scoping reviews to select the included and excluded.

### 2.4. Data Extraction

Data extraction from the articles were on specified characteristics such as country, region, HIV prevalence, other prevalent infectious diseases, availability of CBS, barriers, enablers, and results of implementing CBS.

### 2.5. Data Analysis

The study conducted in-depth analysis of articles related to when case-based surveillance was applied. The researcher aimed to assess the year and time it took to implement the CBS, the approaches and models used. For critical appraisal of individual sources of evidence, we developed a tool aimed at measuring the quality of the article where a score ranged from 0 to 10 with 7 and higher being good quality, 4 to 6 as moderate and 0 to 3 as low quality. The study included all the nine articles which scored good. A decision was made to include all the nine articles scored as good.

### 2.6. Registration of the Scoping Review Protocol

The scoping review was registered with Open Science Framework (OSF), with the registration DOI https://doi.org/10.17605/OSF.IO/VB2J7. The registration details and protocol are publicly available using the internet archive link “https://archive.org/details/osf-registrations-vb2j7-v1 (accessed on 24 January 2026)”. Preferred Reporting Items for Systematic reviews and Meta-Analyses extension for Scoping Reviews (PRISMA-ScR) Checklist can be found in the Supplementary Materials.

## 3. Results

This section discusses the description of the studies, types of models and systems and lastly challenges and successes. For this paper, we reviewed many articles but not all of them were selected for inclusion.

**Figure 1:**
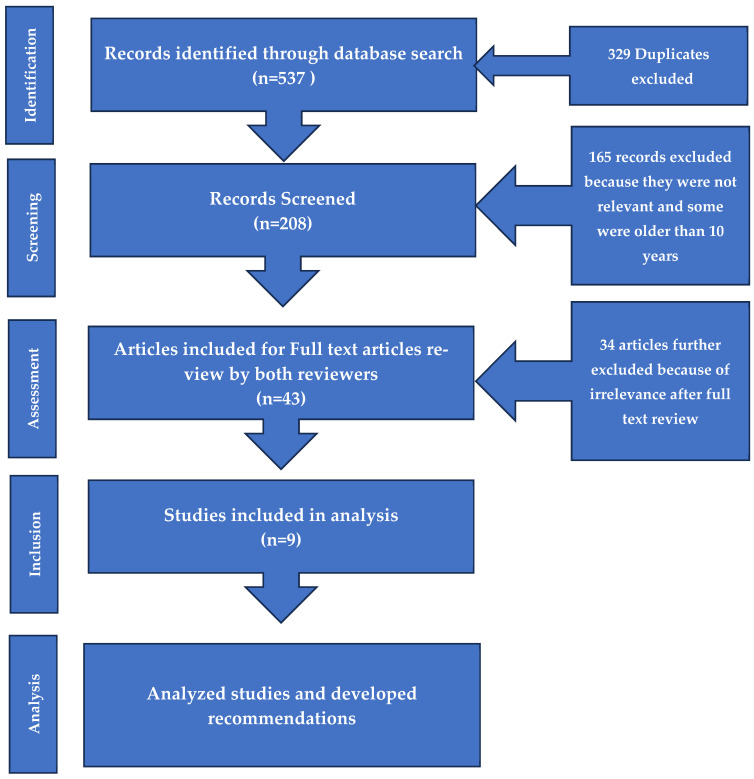
Selection of studies as per PRISMA approach.

Out of 537 publications searched, 329 were duplicates and were therefore excluded. Total articles screened were 208 and about 165 were further excluded because of irreverence and outdated as per inclusion criterion of 10 years. There were 43 articles left as included. The study excluded 34 articles due to insufficient information, some focused on the progress of diseases, not the system, and two full articles could not be accessed. Finally, nine were included and analyzed (Figure 1).

The included articles demonstrated high relevance to CBS, excellent comparison and robust methods [4,5,14]. Also, there was strong emphasis on digital architecture description and assessment, clear CBS operational models and strong data quality assessment relevant to CBS system strengthening [10,15,16,17]. On the other hand, the articles scored moderate and low quality were because of low CBS specificity, moderate depths on CBS functionality and some were just not focused on CBS technical model [8,9,11,18,19].

The graphic or flow diagram below illustrates the articles that were identified, screened, identified as eligible, those included or excluded.

### 3.1. Description of the Studies

This section provides a summary of the description of the studies highlighting the number of studies reviewed, the study designs and study settings. The focus was on countries in the African region although countries outside of Africa were included to assess the global situation pertaining to CBS (Table 1). Some countries were in more than one publication. Of the countries selected, all were in Africa; the majority were in southern Africa (four) [4,5,10,16], two in eastern Africa [4,14], one in western Africa [15] and two in Africa but no specified regions [9,17]. The individual countries mostly focused on South Africa and Zimbabwe in the southern African region [4,16], Rwanda, Tanzania and Kenya in the eastern region and western Africa represented by Guinea. Other countries outside of Africa were Asia and the Caribbean. The studies used several methodological approaches, mixed methods for three studies, one case study, one conceptual Framework, one survey using REDCap, one descriptive cross-sectional study, one combination of in-depth interview and site visit and one combination of desk review, stakeholder meetings and site visits. There were no randomized controlled trials included; the common approaches were cross-sectional and case studies.

### 3.2. Types of Models/Systems

Of the articles reviewed, four focused purely on HIV case-based surveillance while the other five focused on other diseases or public health care (PHC). Eight out of nine articles revealed government ownership of systems for respective countries while one article had no mention of specific ownership. Regarding funding of the systems, 78% were externally funded while the remaining 22% were internally funded by the government or there was no mention of the donors. The U.S. Government through PEPFAR appeared to be the main funder for most of the studies. Malaria surveillance system was also discussed for south Africa and measles was discussed for Zimbabwe and Guinea. Another country in west Africa (Guinea) covered Ebola surveillance but also focused on cholera, meningococcal meningitis, measles, and yellow fever. Academic institutions own most of the publications and very few by donor funded implementing partners (Table 2).

### 3.3. Challenges and Successes

The section reviews and describes a summary of successes and challenges of the CBS systems in Africa.

#### 3.3.1. Successes

Many African countries are responding to the WHO’s call of advocating for a person-centered approach of reporting through case-based surveillance as part of HIV patient monitoring guidelines.

- The studies highlighted the system’s sensitivity as critical and to ensure that there is proper and early detection of public health threats [4,5,15,17].
- Some countries have developed health information systems that are interoperable, which allow for secure data sharing and use which results in better care for patients and eliminates duplication (Rwanda) [14].
- A study conducted in Rwanda showed a successful data exchange between multiple systems including EMR, Lab Information System (LIS), CR and DHIS2 tracker demonstrating a 100% match when generating a dataset for the HIV CBS [14].

#### 3.3.2. Challenges

- Many countries are still struggling with finding the right UID for linking patients to the health services and tracking them in the long-term [4,10].
- Many countries still do not have relevant policies that help ensure data security and confidentiality of patient’s information [4,5].
- Infrastructure continues to be one of the major components hindering successful implementation of CBS especially in rural areas [4,14].
- The use of both paper and electronics continues in many countries with paper-based systems being preferred because of lack of training and dedicated staff for electronic systems. The EMR case study done in Cape Town, South Africa, by [13] confirmed that there is resistance from the clinicians to use full EMR and some hospitals have opted to maintain paper-based systems.

#### 3.3.3. Limitations

There are some limitations which should be considered when applying the study’s conclusions to the Lesotho context. These include the following:

- The studies included used different methodologies, some being descriptive or conceptual, and this might affect comparability and generalization of the results.
- The inaccessibility of some relevant articles might have led to gaps in the review.
- Most reviewed models focus on HIV and certain infectious diseases, which could have left out other health conditions or broader health system factors that influence CBS implementation.

## 4. Discussion

This paper is discussing the different models and systems used in different African countries to assess robust case-based health systems for a stable health system and to determine which ones may be appropriate in a low-income country setting like Lesotho. This research revealed that countries are investing in surveillance systems, especially for infectious diseases, although they are hampered by issues such as inadequate infrastructure and resources, lack of relevant policies, concerns about data security and confidentiality, insufficient capacity and training for staff, data quality, lack of UID and in some cases sensitivity of the systems. Literature review conducted by [16] confirms sensitivity as a challenge with only 61% of malaria cases notified within 24 h in KwaZulu-Natal thereby delaying prompt response for emergencies and outbreaks control. This delay in prompt response is unsatisfactory as per WHO’s recommendations that cases of notifiable diseases including malaria should be reported within 24 h of diagnosis to allow for timely intervention [21]. The use of fingerprints and national ID by some countries are steps in the right direction giving hope to uniquely identify patients [4,5].

Several countries struggle with implementation of UID, which is critical for tracking and linking patients within and across health facilities [10]. The absence of UID also affects data quality and promotes duplication thereby showing a false picture of the prevailing situation. Some countries like South Africa are opting for an algorithm to cater for non-citizens as well instead of using a national ID only [4]. Although the use of national ID poses as a challenge for many countries, a study conducted in Rwanda demonstrated that UID can be implemented within the client registry and be linked with demographic data and other multiple identifiers, which can enable matching across systems even during internet outages [14]. In this study, there was a 100% match between source systems when generating a database for routine HIV CBS; few initial errors were resolved.

There are many factors that contribute to an effective health system such as health financing, health information, health workforce, medical products, leadership and governance. However, a study conducted by Sherr et.al. in five sub-Saharan African countries (Ghana, Mozambique, Rwanda, Tanzania and Zambia) reveals that leadership and governance remained behind and this impedes optimal functioning of the health systems [20].

The use of CBS is imperative for the entire clinical cascade from HIV diagnosis, initiation into antiretroviral therapy (ART), monitoring of disease progression or improvement and finally death [4]. A study conducted in Tanzania, Kenya and South Africa in 2015 which focused on the feasibility of implementing CBS revealed some of the critical elements for CBS including availability of UID which is critical for linking clients’ data within and across health facilities [4].

For countries to have a well-functioning case-based system, establishing an electronic medical record system (EMRs) is one of the first critical steps [8,13]. The study by [13] noted that establishing EMRs is due to increasing infectious diseases that result in death, especially HIV and TB. Thus, Lesotho is on a right track since it is one of the countries that have started developing national EMR systems according to WHO survey [13]. Moreover, the cost of establishing these systems must be a reasonable one, especially for low-income countries. Ref. [13] suggests that open-source systems are ideal as they allow the users to configure them to fit their intended purpose and do not require licensing and software upgrades cost as opposed to expensive propriety systems which may be suitable for wealthier countries.

Although open sources may ensure sustainability, the assessed articles revealed heavy reliance on external funding for establishment and maintenance of the systems. Of the nine articles assessed, only two were internally funded by the government while the all the remaining seven were externally funded particularly by the U.S. Government. Thus, there is need for countries to allocate specific budgets for their health information systems, strengthen local expertise, leverage on domestic resources to cater for HIS and to gradually absorb externally supported system to the local government budget.

Furthermore, regardless of challenges encountered by countries implementing CBS, there is notable progress in Africa. An assessment conducted by CDC in the 46 U.S. President’s Emergency Plan for AIDS Relief (PEPFAR) supported countries to identify enablers and barriers to implementing CBS revealed that “among the 39 (85%) countries that responded, 20 (51%) have implemented CBS, 15 (38%) were planning implementation, and four (10%) had no plans for implementation” [5] (p. 1092). Some of the barriers highlighted by the countries included lack of policies or guidance on CBS, lack of UID and unavailability of data security standards to mention but a few [5].

Thus, Lesotho can learn from the successes and barriers identified by other countries such as avoiding paper-based systems and those that lack integration between health information systems [10]. On the other hand, the country can learn from Tanzania and South Africa that implemented one national system to limit interoperability issues [4]. Also, the availability of dedicated human resources and training, policies for HIV reporting, data security and confidentiality cannot be over-emphasized [4,5,10,13,15]. It is imperative for Lesotho to have UID which will allow for transfer of patient information across facilities [4]. However, in the absence of a national ID as UID, Lesotho can learn from Rwanda by using the client registry and lining it with demographic information and multiple identifiers to enable linkage across facilities [14]. Lesotho, as a low-income African country, is prone to infrastructure challenges especially in the rural areas such as internet and consistent power outages. For a stable surveillance system, the country must invest in these and have contingencies such as generators to allow for a smooth implementation of CBS [4].

Overall, this research highlights the CBS implementation gaps, which Lesotho can avoid when implementing CBS.

## 5. Conclusions

The aim of the study was to review existing models for the case-based health programs in Africa to inform the development and implementation of case-based surveillance of HIV in Lesotho. This research revealed that several African countries have case-based surveillance systems for different health programs such as HIV, malaria, Ebola and measles to mention but a few. Common CBS barriers include limited financial resources, lack of capacity building for staff, lack of relevant policies, limited systems interoperability, data security and lack of confidentiality for patients’ information. For the successful implementation of CBS, countries need to invest in infrastructure, ensure availability of policies, use of open sources, provide capacity building for relevant staff and prioritize use of UID for the ability to track individual patients across health services over time. Until governments cease to rely on donors for HIS support, sustainability and ownership will continue to be hampered.

Based on the objective of the study, tailored recommendations and proposals for Lesotho include adopting and adapting proven models from countries such as South Africa, Kenya and Rwanda, which have established UIDs and interoperable systems. These models emphasize early detection, data accuracy and secure information sharing especially for the HIV high burden country like Lesotho. Also, Lesotho must strengthen infrastructure and capacity building. This includes investing in reliable electricity, internet connectivity and hardware. Similarly, the country must train health workers on digital systems, data management and confidentiality practices which will enhance system acceptance and effectiveness.

Consequently, while external donors can support initial stages of systems’ development, Lesotho needs to plan for domestic funding to ensure sustainability. Also, setting up policies and technical standards that allow different systems to work together is critical. This will make it easier to share data across clinics, labs and the national health database. With these in place, Lesotho will have stable health information systems with better data exchange which will lead to more accurate patient care, quicker responses to outbreaks and stronger overall health management.

## Figures and Tables

**Table 1:** Description of the studies.

	Article Type and Title	Population/Health Program	Study Design/Methods	Country/Region
[16]	Evaluation of the malaria case surveillance system in KwaZulu-Natal Province, South Africa, 2022: a focus on District health Information Software 2 (DHIS2)	Malaria surveillance system	A mixed-method cross-sectional study design	South Africa/southern Africa
[20]	Manuscript: Measuring health systems strength and its impact: experience from the Africa Health Initiative.	General population	Conceptual evaluative framework using World Health Organization’s health systems building block framework	African Health Initiative countries/multi-country
[13]	Electronic Medical Records in low to middle income countries: The case of Khayelitsha Hospital, South Africa.	Trauma cases	Case study evaluating the ability and completeness of the EMR at Khayelitsha hospital to capture all emergencies classified as trauma.	South Africa/southern Africa
[4]	Manuscript: Public Health and Surveillance. Feasibility of Establishing HIV Case-Based Surveillance to Measure Progress Along the Health Sector Cascade: Situational Assessments in Tanzania, South Africa, and Kenya.	HIV Population	A desk review of relevant materials on HIV surveillance and program monitoring, stakeholder meetings, and site visit	Tanzania and Kenya/east Africa South Africa/southern Africa
[5]	Manuscript: Morbidity and Mortality Weekly Report Status of HIV Case-Based Surveillance Implementation—39 U.S. PEPFAR-Supported Countries, May–July 2019.	HIV population HIV CBS	A survey using Research Electronic Data Capture (REDCap), an electronic data management tool hosted at CDC and distributed to each PEPFAR-supported CDC country or regional office	39 PEPFAR supported countries, with majority in Sub-Saharan Africa
[17]	Evaluation of the measles case-based surveillance system in Kwekwe city, 2017–2020: descriptive cross-sectional study	General population Measles CBS	Descriptive cross-sectional study using CDC surveillance guidelines	Zimbabwe/southern Africa
[15]	Surveillance system assessment in Guinea: Training needed to strengthen data quality and analysis, 2016	General Population Case and community-based surveillance for: cholera, meningococcal meningitis, measles, and yellow fever	In-depth interviews with key informants and site visits	Guinea/west Africa
[10]	Progress towards unique patient identification and case-based surveillance within the Southern African development community	HIV population CBS with a unique patient identification (UPI)	Mixed-method landscape analysis of UPI and CBS implementation	Southern African Development Community (SADC) countries
[14]	Implementation of an HIV Case Based Surveillance Using Standards-Based Health Information Exchange in Rwanda	HIV population CBS for HIV implementation	Quasi experimental, mixed methods	Rwanda/east Africa

**Table 2:** Models of CBS in Africa.

	Disease Focus	Source of Funding	Countries	Model/System/Owner
[16]	Malaria	Internal: National Department of Health; no external donors mentioned	South Africa	Evaluation: Malaria case surveillance system: DHIS2 The systems in this article are DHIS2 which is used as central data management systems and malaria case surveillance flow system which supports tracking case classifications and ensures timely reporting of cases. Data are collected by health care workers at facility level into Malaria surveillance system and then integrated into DHIS2 either manually or automatically. Both systems are owned by the Government of South Africa although managed by other partners. The four critical components of a surveillance system are data quality, timeliness, simplicity, and acceptability An effective surveillance system is critical in evaluating the plans to achieve elimination Although data quality was generally accepted, timeliness of reporting cases within 24 h remained a challenge For optimum use and acceptability of the systems, giving feedback to lower surveillance levels is crucial
[20]	This article is not disease based but focused on population health with focus on child mortality	External: Doris Duke Charitable Foundation funded this study	Ghana, Mozambique, Rwanda, Tanzania, and Zambia	Evaluation framework to measure health systems strength Assessing association between health systems measures and health outcomes. Six WHO core blocks measured were service delivery, Health workforce, information systems, medical products, vaccines and technologies, health financing and leadership and guidance. There were some attributes of health systems that could not be evaluated, and these include trust, resilience, quality, and leadership. The six WHO health systems are limited in measuring validity, sensitivity and comprehensive metrics of health systems. Effective evaluation of health systems strength requires sophisticated evaluation methods, indicators in context and understanding how various systems work.
[13]	This study focuses on trauma cases	External: The study was externally funded by Down’s Fellowship and Yale School of Medicine, but the donors of the system are not mentioned.	South Africa	Electronic Medical Records system. The assessment at KH was used as a proxy which would reflect nationwide estimates of about 40% of emergency center visits. KH is using both Enterprise Content Management (ECM) and EMR. The systems were deployed in 2012 and are owned by the government although they are controlled by JAC Computer services because they are proprietary systems. Patient’s data are collected at the hospital through EMR, ECM and the file. For a successful electronic medical record system, funding must be secured for adequate training and supervision of users and other necessary resources Adequate records system is a pillar of the health facility without which it is prone to collapsing
[4]	Focused on HIV	External: The article was externally funded by Bill and Melinda Gates Foundation, WHO and Global Fund to Fight AIDS, Tuberculosis, and Malaria but the systems are public or government owned.	Tanzania, South Africa, and Kenya.	Situational Assessment: Case-base surveillance All systems are owned by the government In Tanzania, data are collected at individual-level from point of entry into care on approximately two-thirds of people on ART. In SA, the system collects individual-level data at the facility and then reported to the national level including names and other personal identifiable factors. In Kenya, EMRs are used for facilities with patients greater than 500. Individual-level data are captured in the EMR, and aggregate data are reported to the central data warehouse on quarterly basis. The systems, though funded externally, are owned by the government in respective countries. All three countries do not have policies for HIV reporting, data security and confidentiality. The only policy in SA is for vital registration data and Kenya has some policy for infectious diseases but not specific to HIV. In Tanzania and Kenya, de-duplication of patients’ data is done using clinical identifier while SA uses an algorithm All the three countries reported internet challenges in the rural areas. Tanzania thought of interoperability as unnecessary because the PMS database is national. SA on the other hand uses Tier.Net which is also a national system therefore limiting interoperability issues. Kenya has 4 EMRs that are not interoperable and data in each system have not been evaluated.
[5]	The disease focus in this article was HIV	External: The study was funded by the United States Government through CDC/PEPFAR program	Angola, Botswana, Brazil, Cambodia, Côte d’Ivoire, Democratic Republic of the Congo, Dominican Republic, El Salvador, Eswatini, Ethiopia, Ghana, Guatemala, Guyana, Haiti, Honduras, Jamaica, Kenya, Laos, Lesotho, Mali, Malawi, Mozambique, Namibia, Nicaragua, Nigeria, Panama, Papua New Guinea, Rwanda, Senegal, South Africa, South Sudan, Tanzania, Thailand, Trinidad and Tobago, Uganda, Ukraine, Vietnam, Zambia, and Zimbabwe	CBS implementation assessment Of the 20 countries implementing CBS, all collect date of HIV diagnosis and 85% collect sentinel event survey data and 50% of these countries use the UID to link and de-duplicate patients’ data. Countries already implementing CBS and those planning to implement have funding, mostly from PEPFAR and they have dedicated human resource for the systems. Of the 39 countries assessed, 20 had already implemented CBS, 15 were planning to 4 were not planning to implement Challenges reported especially in Sub-Saharan Africa included lack of UID limiting data linkage across systems and lack of national policies and data security standards. The 4 countries that were not planning to implement CBS indicated lack of funding and dedicated human resources as major barriers.
[17]	Measles	Internal: This is a public health surveillance and there is no mention of external donors.	Zimbabwe	Descriptive cross-sectional assessment using CDC guidelines for surveillance system evaluation. The measles CBS in Zimbabwe is a government-owned system integrated with other vaccine preventable diseases such as acute flaccid paralysis. Data for all suspected cases of measles are routinely collected at all levels of health delivery using measles case surveillance form. Data from primary health facilities are sent to the district, then to the province and finally to the national level. This system is owned by the local Department of Health. The evaluation revealed that although most users confirmed that the CBS was simple, it lacked stability, acceptability and sensitivity. Lack of training was shown as one of problems for underperformance of measles CBS. Also, lack of relevant staff for the system hindered its optimum use. Engagement of relevant stakeholders such as private sector and the community is key for the success of the system.
[15]	The assessment focused on four diseases, namely cholera, meningococcal meningitis, measles and yellow fever	External: the study was funded by the US Government through CDC but is a government-owned public health surveillance system.	Guinea	Surveillance system assessment using CDC’s guidelines for surveillance system evaluation. This is a government-owned system supported by international partners. The assessment was focused on the surveillance system’s operations, resources, and attributes particularly simplicity and data quality. At health-center level, the surveillance system is paper-based while at prefectural and central levels, it is computer spreadsheet-based. The Ministry of Health surveillance protocol required immediate and routine weekly reporting at health and prefectural levels and then reported at central by telephone. This is a public health system owned by the government in Guinea The assessment revealed that the system in Boffa was simple but had limitations in documentation and data analysis. The Ebola outbreak in 2014–2016 revealed Guinea’s weak health systems and surveillance gaps hindering proper detection and swift response to emerging disease outbreaks. The system’s sensitivity was determined as low as no cases or the four diseases were identified during the assessment period although data suggested existence of cases. For a successful surveillance system, the country needs to improve capacity building for the users, improve infrastructure such as electricity and enhance feedback mechanisms to encourage data analysis and use.
[10]	The assessment focuses on HIV although there is mention of hypertension, diabetes, and tuberculosis (TB).	External: the assessment was funded though PEPFAR, and there is strong emphasis strong US support to health information systems.	Botswana, Eswatini, Lesotho, Mozambique, Namibia, South Africa, Zambia and Zimbabwe	Landscape analysis of unique patient identification (UPI) and CBS implementation within selected SADC countries. The commonly collected identifiers are patient name, date of birth, government ID, phone numbers and facility file number. The system is owned by the government through the Ministry of Health in all the countries respectively. UPI implementation is limited by paper-based systems and lack of integration between health information systems. Many countries still rely on paper-based systems and fragmented electronic systems that are not integrated. Common CBS barriers include limited financial resources, lack of capacity building for staff, limited systems interoperability, data security and lack of confidentiality for patients’ information. Most SADC countries are in the early to middle stages of developing patient-centered, case-based surveillance systems using UPIs.
[14]	Disease focus is HIV	External: The HIV CBS in Rwanda is particularly PEPFAR-funded	Rwanda	Assessed health information exchange ecosystem focusing on open sources and standards supporting generation of complete data sets needed for HIV CBS in Rwanda. The systems are owned by the government but financially supported by PEPFAR. Data collection is done at health center level and collects patient-level data. The study revealed that using open sources such as HL7 FHIR is effective and enables interoperability of systems in low-resource settings. In the absence of national ID as UID, the study demonstrated that UID can be done with client registry linking it with demographic data and multiple identifiers to enable linkage and matching across different systems.

## Data Availability

All articles reviewed are publicly available via links within the paper.

## Supplementary Materials

The following supporting information can be downloaded at: https://www.mdpi.com/article/10.3390/ijerph23030308/s1, File S1: Preferred Reporting Items for Systematic reviews and Meta-Analyses extension for Scoping Reviews (PRISMA-ScR) Checklist [22].

## Abbreviations

The following abbreviations are used in this manuscript:

**Acronym**	**Definition**
CBS	Case-Based Surveillance
EMRs	Electronic Medical Records
PLHIV	People Living with HIV
ART	Antiretroviral Treatment
UID	Unique Identification Number
WHO	World Health Organization
PEPFAR	President’s Emergency Plan for AIDS Relief
LIS	Laboratory Information System
CR	Client Registry
DHIS2	District Health Information System 2
REDCap	Research Electronic Data Capture
ECM	Enterprise Content Management
PMS	Patient Monitoring System
HL7 FHIR	Health Level Seven Fast Healthcare Interoperability Resources
SOPs	Standard Operating Procedures
SADC	Southern African Development Community
GPA	Global Program on AIDS
CDC	Centers for Disease Control and Prevention
EBSCOHOST	A research database platform
PRISMA	Preferred Reporting Items for Systematic Reviews and Meta-Analyses
COVID-19	Coronavirus Disease 2019
STI	Sexually Transmitted Infection
ECM	Enterprise Content Management (also listed above)
N/A	Not Applicable
