# Supplementary material for: Scoping Review of the Models for Case-Based Health Programs in Africa: Towards Case-Based Surveillance for HIV in Lesotho
Source: Int J Environ Res Public Health. 2026 Feb 28;23(3):308. doi: 10.3390/ijerph23030308 (PMC13026963; doi:10.3390/ijerph23030308)
Supplement: Supplementary file 1 [file ijerph-23-00308-s001.zip › ijerph-4046472-supplementary.pdf]

Preferred Reporting Items for Systematic reviews and Meta-Analyses extension for Scoping Reviews (PRISMA-ScR) Checklist

SECTION	ITEM	PRISMA-ScR CHECKLIST ITEM	REPORTED ON PAGE #
**TITLE**			
Title	1	Identify the report as a scoping review.	Scoping review of the models for case-based health programs in Africa **(P1)**
**ABSTRACT**			
Structured summary	2	Provide a structured summary that includes (as applicable): background, objectives, eligibility criteria, sources of evidence, charting methods, results, and conclusions that relate to the review questions and objectives.	The review was aimed at exploring models across Africa that could best help Lesotho succeed in its efforts to establish a case-based surveillance (CBS) system for their HIV pro-gram. Research involved looking through several sources and databases including EB-SCOHOST, Google Scholar, Science Direct and PubMed. The insights of suitable models were from the following Africa countries: South Africa, Kenya, Guinea, Tanzania, Ghana, Mozambique and Zambia. The researched models focused on infectious diseases such as measles, HIVand COVID-19. The key takeaway is that setting up electronic medical records systems (EMRs) is critical as a first step for any effective CBS. Also, using unique identifiers, establishing clear data governance policies and building strong infrastructure is a necessity in making CBS work. For a successful establishment of CBS, Lesotho should adopt these strategies that can be sustainable, improve disease tracking, response and ultimately health outcomes for Basotho.<br>Keywords: HIV and surveillance, case-based surveillance, patient-level health systems, infectious diseases surveillance, and Africa **(P2)**
**INTRODUCTION**			
Rationale	3	Describe the rationale for the review in the context of what is already known. Explain why the review questions/objectives lend themselves to a scoping review approach.	Although various models and systems for case-based health surveillance exist across Africa, there is limited comprehensive synthesis and understanding of their design, implementation, successes, and challenges. Existing literature indicates that while some countries have made strides in establishing surveillance systems, particularly for HIV and other infectious diseases. These efforts are often fragmented, with considerable variability in system ownership, technological infrastructure, and operational capacity. Moreover, there is a lack of consolidated knowledge on how different models function within diverse resource settings, and what factors influence their sustainability and effectiveness. The review questions and objectives are well-suited to a

St. Michael's
Inspired Care.
Inspiring Science.

SECTION	ITEM	PRISMA-ScR CHECKLIST ITEM	REPORTED ON PAGE #
			scoping review approach because they involve exploring a broad and complex field characterized by diverse system designs, implementation contexts, and stakeholder perspectives. **(P2&3)**
Objectives	4	Provide an explicit statement of the questions and objectives being addressed with reference to their key elements (e.g., population or participants, concepts, and context) or other relevant key elements used to conceptualize the review questions and/or objectives.	1) To identify and describe the existing models for case-based health programs in Africa.<br>2) To determine successes and challenges to implementation of these models in Africa.<br>3) To assess the needs for development and implementation of a case-based surveillance and to recommend a model that could be relevant for Lesotho.<br>4) To identify any gaps in the models that could be considered when developing a case-based surveillance for HIV in an African setting. **(P3)**
**METHODS**			
Protocol and registration	5	Indicate whether a review protocol exists; state if and where it can be accessed (e.g., a Web address); and if available, provide registration information, including the registration number.	The scoping review has been registered with open science Framework. The assigned doi is: https://doi.org/10.17605/OSF.IO/VB2J7 and the internet Archive Link is https://archive.org/details/osf-registrations-vb2j7-v1
Eligibility criteria	6	Specify characteristics of the sources of evidence used as eligibility criteria (e.g., years considered, language, and publication status), and provide a rationale.	The inclusion criterion focused on articles related to implementation of case-based surveillance for infectious diseases not older than 10 years (2014-2024), documents written in English, and both quantitative and qualitative studies to assess the differences and results when implementing CBS. **(P4)**
Information sources*	7	Describe all information sources in the search (e.g., databases with dates of coverage and contact with authors to identify additional sources), as well as the date the most recent search was executed.	There were several databases that were used to search for articles, and these included EBSCOHOST, Science Direct and PubMed to mention but a few. The following key words were used to search for documents: HIV and surveillance, case-based surveillance, patients-level health systems, infectious diseases surveillance and Africa. For this paper, many articles were reviewed but not all of them were selected for inclusion. **(P4)**
Search	8	Present the full electronic search strategy for at least 1 database, including any limits used, such that it could be repeated.	On PubMed, the researcher went to the website and put in the key words and the years preferred. Relevant studies were identified and included. **(P4)**
Selection of sources of evidence†	9	State the process for selecting sources of evidence (i.e., screening	Data extraction from the articles was based on specified characteristics such as country, region, HIV prevalence, other prevalent

St. Michael's
Inspired Care.
Inspiring Science.

SECTION	ITEM	PRISMA-ScR CHECKLIST ITEM	REPORTED ON PAGE #
		and eligibility) included in the scoping review.	infectious diseases, availability of CBS, barriers, enablers, and results of implementing CBS. **(P6)**
Data charting process‡	10	Describe the methods of charting data from the included sources of evidence (e.g., calibrated forms or forms that have been tested by the team before their use, and whether data charting was done independently or in duplicate) and any processes for obtaining and confirming data from investigators.	Two reviewers assessed the articles and provided feedback in Ryyan. Conflicts were resolved through a meeting, and final articles were selected. **(P4)**
Data items	11	List and define all variables for which data were sought and any assumptions and simplifications made.	Data abstraction from the articles was based on specified characteristics such as country, region, HIV prevalence, other prevalent infectious diseases, availability of CBS, barriers, enablers, and results of implementing CBS. **(P4)**
Critical appraisal of individual sources of evidence§	12	If done, provide a rationale for conducting a critical appraisal of included sources of evidence; describe the methods used and how this information was used in any data synthesis (if appropriate).	Many countries are lagging with implementation of CBS for HIV programs. Thus, in-depth assessment of the methods applied to develop the system was explored. The researcher also aimed at assessing the year and time it took to implement the CBS, the approaches and models used. **(P3)**
Synthesis of results	13	Describe the methods of handling and summarizing the data that were charted.	Data synthesis was done to group the studies and summarize based on region, study designs and methods, models used and general findings. **(P6)**
**RESULTS**			
Selection of sources of evidence	14	Give numbers of sources of evidence screened, assessed for eligibility, and included in the review, with reasons for exclusions at each stage, ideally using a flow diagram.	. Out of 537 publications searched, 329 were duplicate and therefore excluded. Total articles screened were 208 and about 182 were further excluded because of irreverence and outdated as per inclusion criterion of 10 years. There were 43 articles left as included. The 34 articles that were excluded were due to insufficient information, some were focusing on the progress of diseases, not the system and two (2) full articles could not be accessed. Finally, nine (9) were included and analyzed. **(P5)**
Characteristics of sources of evidence	15	For each source of evidence, present characteristics for which data were charted and provide the citations.	The focus was on countries in the African region although countries outside of Africa were included to assess the global situation pertaining to CBS (Table 1 of the manuscript). Some countries were included in more than one publication. Of the countries selected, in

SECTION	ITEM	PRISMA-ScR CHECKLIST ITEM	REPORTED ON PAGE #
			Africa; majority were Southern Africa (four (4)), two (2) in Eastern Africa, one (1) in Western Africa and two (2) in Africa but not specified regions. The individual countries mostly focused on South Africa and Zimbabwe in the Southern African region, Rwanda, Tanzania and Kenya in the Eastern region and Western Cape is represented by Guinea. Other countries outside of Africa were Asia and the Caribbean. **(P6&7)**
Critical appraisal within sources of evidence	16	If done, present data on critical appraisal of included sources of evidence (see item 12).	All the articles were according to the eligibility criteria and dealt with infectious diseases. They had all developed CBS. Of the articles reviewed, four (4) focused purely on HIV case-based surveillance while the other five (5) focused on other diseases or public health care (PHC). Eight (8) out of nine (9) articles revealed government ownership of systems for respective countries while one (1) article had no mention of specific ownership. **(P9)**
Results of individual sources of evidence	17	For each included source of evidence, present the relevant data that were charted that relate to the review questions and objectives.	Eight (8) out of nine (9) articles revealed government ownership of systems for respective countries while one (1) article had no mention of specific ownership. Regarding funding of the systems, 78% (7/9) were externally funded while the remaining 22% (2/9) were internally funded by the Government or there was no mention of the donors. The U.S. Government through PEPFAR appeared to be the main funder for most of the studies. Malaria surveillance system was also discussed for South Africa and measles was discussed for Zimbabwe and Guinea. Another country in West Africa (Guinea) covered Ebola surveillance but also focused on cholera, meningococcal meningitis, measles, and yellow fever. Majority of the publications were owned by academic institutions and very few by donor funded implementing partners (Table 2 of the manuscript). **(P9)**
Synthesis of results	18	Summarize and/or present the charting results as they relate to the review questions and objectives.	This research revealed that countries are investing in surveillance systems, especially for infectious diseases, although they are hampered by issues such as inadequate infrastructure and resources, lack of relevant policies, concerns about data security and confidentiality, insufficient capacity and training for staff, data quality, lack of UID and in some cases sensitivity of the systems. The absence of UID also affects data quality and promotes duplication thereby showing a false picture of the prevailing situation. Some countries like South Africa are opting for an algorithm to cater for non-citizens as well instead of using a

SECTION	ITEM	PRISMA-ScR CHECKLIST ITEM	REPORTED ON PAGE #
			national ID only. **(P19)**
**DISCUSSION**			
Summary of evidence	19	Summarize the main results (including an overview of concepts, themes, and types of evidence available), link to the review questions and objectives, and consider the relevance to key groups.	The aim of the study was to review existing models for the case-based health programs in Africa to inform the development and implementation of case-based surveillance of HIV in Lesotho. This research revealed that several African countries have case-based surveillance systems for different health programs such as HIV, Malaria, Ebola and measles to mention but a few. Common CBS barriers include limited financial resources, lack of capacity building for staff, lack of relevant policies, limited systems interoperability, data security and lack of confidentiality for patients' information. For the successful implementation of CBS, countries need to invest in infrastructure, ensure availability of policies, use of open sources, provide capacity building for relevant staff and prioritize use of UID for the ability to track individual patients across health services over time. Until governments cease to rely on donors for HIS support, sustainability and ownership will continue to be hampered. **(P19)**
Limitations	20	Discuss the limitations of the scoping review process.	There were several limitations of the scoping review which included limited articles that were more recent. Many articles were over 10 years and therefore could not be included. There were other articles that were not free and not be accessed even through the university library. **(P4)**
Conclusions	21	Provide a general interpretation of the results with respect to the review questions and objectives, as well as potential implications and/or next steps.	This research revealed that several African countries have case-based surveillance systems for different health programs such as HIV, Malaria, Ebola and measles to mention but a few. Common CBS barriers include limited financial re-sources, lack of capacity building for staff, lack of relevant policies, limited systems interoperability, data security and lack of confidentiality for patients' information. For the successful implementation of CBS, countries need to invest in infrastructure, ensure availability of policies, use of open sources, provide capacity building for relevant staff and priori-tize use of UID for the ability to track individual patients across health services over time. Until governments cease to rely on donors for HIS support, sustainability and ownership will continue to be hampered. **(P20)**
**FUNDING**			
Funding	22	Describe sources of	This research received no external funding, the

SECTION	ITEM	PRISMA-ScR CHECKLIST ITEM	REPORTED ON PAGE #
		funding for the included sources of evidence, as well as sources of funding for the scoping review. Describe the role of the funders of the scoping review.	researcher only received tuition assistance from the University of the Free State but there was no specific funding for this scoping review. **(P20)**

JBI = Joanna Briggs Institute; PRISMA-ScR = Preferred Reporting Items for Systematic reviews and Meta-Analyses extension for Scoping Reviews.

* Where *sources of evidence* (see second footnote) are compiled from, such as bibliographic databases, social media platforms, and Web sites.

† A more inclusive/heterogeneous term used to account for the different types of evidence or data sources (e.g., quantitative and/or qualitative research, expert opinion, and policy documents) that may be eligible in a scoping review as opposed to only studies. This is not to be confused with *information sources* (see first footnote).

‡ The frameworks by Arksey and O'Malley (6) and Levac and colleagues (7) and the JBI guidance (4, 5) refer to the process of data extraction in a scoping review as data charting.

§ The process of systematically examining research evidence to assess its validity, results, and relevance before using it to inform a decision. This term is used for items 12 and 19 instead of "risk of bias" (which is more applicable to systematic reviews of interventions) to include and acknowledge the various sources of evidence that may be used in a scoping review (e.g., quantitative and/or qualitative research, expert opinion, and policy document).

From: Tricco AC, Lillie E, Zarin W, O'Brien KK, Colquhoun H, Levac D, et al. PRISMA Extension for Scoping Reviews (PRISMAScR): Checklist and Explanation. Ann Intern Med. 2018;169:467–473. doi: 10.7326/M18-0850.
